# Comparative transcriptome analysis of resistant and susceptible kiwifruits in response to *Pseudomonas syringae* pv. *Actinidiae* during early infection

**DOI:** 10.1371/journal.pone.0211913

**Published:** 2019-02-19

**Authors:** Yalin Song, Leiming Sun, Miaomiao Lin, Jinyong Chen, Xiujuan Qi, Chungen Hu, Jinbao Fang

**Affiliations:** 1 Zhengzhou Fruit Research Institute, Chinese Academy of Agricultural Sciences, Zhengzhou, China; 2 College of Horticulture and Forestry Sciences, Huazhong Agricultural University, Wuhan, China; Key Laboratory of Horticultural Plant Biology (MOE), CHINA

## Abstract

Kiwifruit bacterial canker is a devastating disease threatening kiwifruit production. To clarify the defense mechanism in response to *Pseudomonas syringae* pv. *actinidiae* (Psa), we observed phenotypic changes in resistant Huate (HT) and susceptible Hongyang (HY) kiwifruit varieties at 0, 12, 24, 48, 96, and 144 hour after inoculation (hai) with Psa. Brown lesions appeared in the inoculation areas 12 hai in HY shoots, and the lesion length gradually increased from 24 to 144 h. In contrast, no lesions were found in HT shoots at any time points. Furthermore, RNA-seq analysis showed significantly more differentially expressed genes between HT and HY at 12 hai than at any other time point. According to weighted gene co-expression network analysis, five modules were notably differentially expressed between HT and HY; pathway mapping using the Kyoto Encyclopedia of Gene and Genomes database was performed for the five modules. In MEgreenyellow and MEyellow modules, pathways related to“plant-pathogen interaction”, “Endocytosis”, “Glycine, serine and threonine metabolism”, and “Carbon fixation in photosynthetic organisms” were enriched, whereas in the MEblack module, pathways related to “protein processing in endoplasmic reticulum”, “plant-pathogen interaction”, and “Glycolysis / Gluconeogenesis” were enriched. In particular, the *Pti1* and *RPS2* encoding effector receptors, and the *NPR1*, *TGA*, and *PR1* genes involved in the salicylic acid signaling pathway were significantly up-regulated in HT compared with HY. This indicates that the effector-triggered immunity response was stronger and that the salicylic acid signaling pathway played a pivotal role in the Psa defense response of HT. In addition, we identified other important genes, involved in phenylpropanoid biosynthesis and Ca^2+^ internal flow, which were highly expressed in HT. Taken together, these results provide important information to elucidate the defense mechanisms of kiwifruit during Psa infection.

## Introduction

Bacterial canker caused by *Pseudomonas syringae* pv. *actinidiae* (Psa) is a devastating disease that affects kiwifruit (*Actinidia*). The disease can cause the death of kiwifruit trees in a large area with its fast transmission and strong pathogenicity. It has caused serious yield and economic losses in many countries and has become a major limiting factor in the development of the kiwifruit industry [1,2]. In New Zealand, kiwifruit bacterial canker disease became a great threat to the local kiwifruit industry, in November 2010, wherein a large number of kiwifruit orchards were confirmed to be infected with Psa (http://www.kvh.org.nz/). The disease’s incidence and infection patterns are consistent throughout the world. Psa enters the vine through openings like the stomata and lenticels, and through wounds caused by insects, wind, and rain. The symptoms include milky white bacteria which exudes from the trunk and cane; the cane phloem turning brown; irregular or polygonal brown spots on the leaves, resulting in the development of lesions with a 3–5 mm yellow halo; and buds that wither after Psa infection [3,4].

The occurrence of kiwifruit bacterial canker is closely related to the climate. The rain, high humidity, and low temperatures (12°C-18°C) in Italy are particularly conducive to the propagation and rapid spread of Psa [5]. In addition, kiwifruits are infected with Psa on a large scale in a short time, which is inseparable from its infection principles. Psa can degrade lignin and phenols, which is conducive to its growth [6], and can effectively absorb iron ions from the plants to enhance its virulence [7,8]. The current methods of preventing kiwifruit bacterial canker include chemical and biological controls. At present, streptomycin and copper agent are commonly used. However, some studies reported that the pathogen can develop resistance to pesticides and antibiotics. Psa initially contained only two anti-copper factors, *copA* and *copB*. However, after prolonged use of high copper preparations for prevention and treatment, the pathogen produced two new anti-copper factors, *copR* and *copS* [9].

There are significant differences in resistance to bacterial canker disease between species and cultivars. From 2008 to 2011, most of the gold kiwifruit orchards in Italy were destroyed because of the high susceptibility of the cultivars, including Hort16A and Jin Tao. Psa also infects green kiwifruit like the Hayward in many regions of Italy [10]. New Zealand researchers found that Hongyang and Hort16A were highly susceptible to Psa, while Green14 was resistant to Psa (http://www.kvh.org.nz/). In addition, Jinkui and Kuimi were disease-resistant varieties, whereas Qinmei and Jinfeng were susceptible varieties in Anhui, China [11]. The pathogen has also been isolated from both gold kiwifruit and green kiwifruit in China. Our research group previously identified kiwifruit bacterial canker in the Hongyang (HY) and Huate (HT) varieties. The results showed that HY is a highly susceptible variety, while HT is a highly resistant variety. It is critical to clarify the molecular resistance mechanisms of kiwifruit plants and breed new cultivars with high resistance.

Through evolution, plants can form a series of complex adaptation mechanisms against pathogens. The plant innate immune system is divided into two aspects: Pathogen-associated molecular pattern-triggered immunity (PTI) and effector-triggered immunity (ETI) [12–14]. PTI is induced by the recognition of pathogen-associated molecular patterns through pattern recognition receptors at the plant cell surface, which activates a mitogen-activated protein kinase cascade that results in defense responses [15,16]. Pathogens can evolve a series of effectors that inhibit recognition by the host cells; however these can interact with resistance proteins to initiate the second stage of the plant immune system: ETI. ETI activates the plant’s hypersensitivity response, which prevents the spread of pathogens to adjacent healthy tissue and causing plant systemic acquired resistance [17]. Acibenzolar-S-methyl, a functional analogue of salicylic acid (SA), is one of the most effective elicitors for Psa control [18]. Previous studies in the model plant *Arabidopsis thaliana* have identified resistance-related genes and proteins that promote the recognition of plant-pathogen interactions after infection with Psa in kiwifruit plants [19,20].

In this study, we conducted transcriptome sequencing analysis of the resistant kiwifruit cultivar HT and the susceptible cultivar HY after infection with Psa. We analyzed differentially expressed genes (DEGs) after Psa infection, and discussed the possible factors causing kiwifruit bacterial canker. The results could help in exploring the resistance-genes and biological pathways associated with the kiwifruit bacterial canker disease, and in understanding the molecular mechanisms of kiwifruit plants’ defenses against Psa.

## Materials and methods

### Plant materials and Psa inoculation

*Actinidia eriantha* Bentham cv HT and *A*. *chinensis* Planchon cv HY were grown in the *Actinidia* germplasm resources repository of the Zhengzhou Fruit Research Institute, Chinese Academy of Agricultural Sciences, located in Zhengzhou, Henan province, China. HT has been proven to be resistant to Psa while HY is susceptible to it. One-year old shoots, approximately 0.8 cm in diameter, were collected from the vines in April 2017. The pathogen Psa was provided by the Zhejiang Academy of Agricultural Sciences. Psa was cultured on beef peptone medium for 24 h at 20°C. The microbial concentration of Psa was diluted to 10^8^ colony-forming units (cfu)/ml prior to inoculation. For inoculation, the detached shoots were surface sterilized with chlorine and then cut into 10 cm shoots and the ends of the shoots were dipped in candle wax to reduce dehydration. A wound was made with a file about 1–1.5 cm from each end of the shoot and Psa was added to the wound with a pipette. Control shoots were treated with sterile water. The inoculated and control shoots were placed in trays, which were placed in an artificial climate incubator at 20°C and 80% relative humidity for 12 h day/night cycles.

Shoot samples were taken from the inoculated and mock-inoculated segments 0.5–1 cm away from the wound point at 0, 12, 24, 48, and 96 hai. The samples were immediately placed in liquid nitrogen and stored at -80°C for RNA extraction and further analysis. Each of the fifteen samples contained six shoots from three different vines, as biological replicates.

### Total RNA extraction, cDNA library construction, and sequencing

Total RNA was extracted using the RNA prep Pure Plant Kit (Polysaccharides & Polyphenolics-rich), following the manufacturer’s protocol. RNA concentration was measured using the Qubit RNA Assay Kit in a Qubit 2.0 Flurometer (Life Technologies, CA, USA) and RNA integrity was assessed using the RNA Nano 6000 Assay Kit of the Bioanalyzer 2100 system (Agilent Technologies, CA, USA). Following RNA quantification and qualification, mRNA was purified from total RNA using poly-T oligo-attached magnetic beads and the mRNA was then broken into fragments using a fragmentation buffer. First strand cDNA was synthesized using random hexamer primers, and second strand cDNA synthesis was subsequently performed using buffer, dNTPs, DNA Polymerase I, and RNase H. The library fragments were purified with AMPure XP beads (Beckman Coulter, Beverly, USA), and USER enzyme was used with size-selected, adaptor-ligated cDNA before PCR. PCR was performed to enrich the purified cDNA libraries. Finally, the library preparations were sequenced on an Illumina HiSeq platform.

### Sequencing read mapping and identification of DEGs

Raw reads in FASTQ format were generated by base calling. Clean reads were obtained by removing reads with adapters, reads containing more than 10% ploy-N (where N refers to unknown bases), and low-quality reads. All subsequent analyses were based on the clean data, which were aligned to the reference genome using the TopHat v2.0.12 [21]. Gene expression levels were calculated using the FPKM method (expected number of fragments per kilobase of transcript sequence per millions of base pairs sequenced) using HTSeq v0.6.1 [22]. DEGs were analyzed using the DESeq R package (1.18.0) [23]. The P-values were adjusted using Benjamini and Hochberg’s approach for controlling the false discovery rate. Genes with an adjusted P-value <0.05 found by DESeq were assigned as differentially expressed [24].

### Gene ontology and KEGG pathway analysis

Gene ontology (GO) enrichment analysis of DEGs was implemented using the GOseq R package [25]. Gene ontology terms with corrected P<0.05 were considered significantly enriched in DEGs. KOBAS software was used to test the enrichment of DEGs in KEGG (Kyoto Encyclopedia of Gene and Genomes) pathways [26].

### Gene co-expression network analysis

Weighted gene co-expression network analysis is a common algorithm for constructing gene co-expression networks [27]. DEGs (P<0.05) were analyzed in all samples. Gene dendrograms were constructed with colors based on the correlations between the expression of genes. These were used to build clustering trees, and to divide the modules. In addition, the correlation between modules and samples was also analyzed by weighted gene co-expression network analysis.

### Quantitative real-time PCR analysis

To verify the RNA-Seq analysis, we designed primers for qRT-PCR using the vector NTI. Template cDNA was synthesized using a qPCR-RT Kit (TOYOBO) and the qRT-PCR was performed on a RT-PCR instrument (Roche 480, Basel, Switzerland). The 2×SYBR Green I RT-PCR Master Mix (Roche) was used as a fluorescent reporter. The reaction was performed with the following program: pre-incubation for 5 min at 95°C, followed by 40 cycles of amplification of 10 s at 95°C, 20 s at 60°C, and 20 s at 72°C. The relative gene expression levels were calculated using the 2^-ΔΔc(t)^ method.

## Results and discussion

### Changes in symptoms of HT and HY shoots infected with Psa

**Fig 1:**
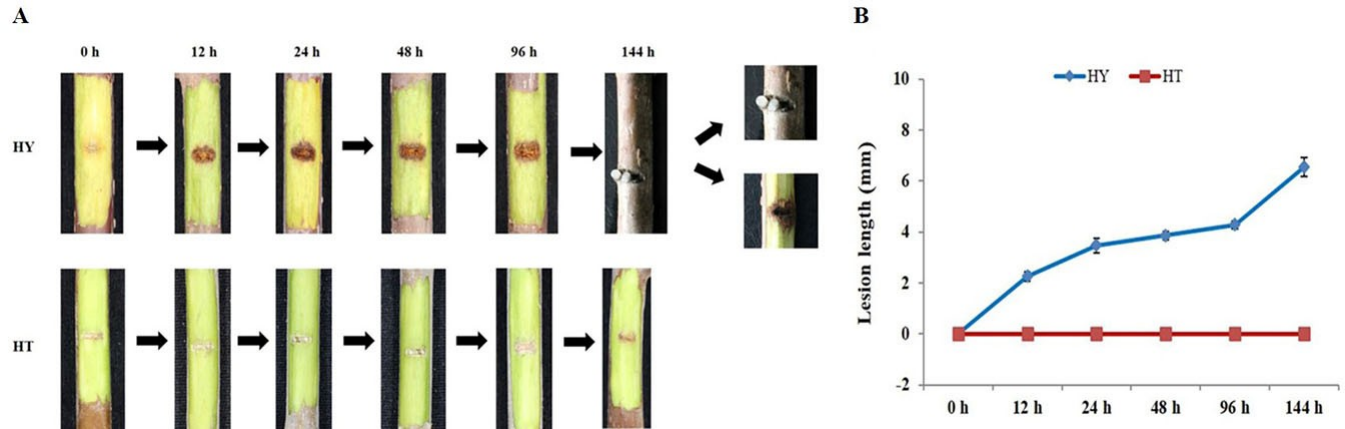
Disease symptoms in Huate (HT) and Hongyang (HY) at 0, 12, 24, 48, 96, and 144 hai with Psa. (A) Photos of the phenotypic changes. (B) Changes in lesion length at 0, 12, 24, 48, 96, and 144 hai with Psa. The values presented are the means ± SD of six replicates of HT and HY at 0, 12, 24, 48, 96, and 144 hai with Psa.

There are significant differences in resistance to Psa among infected kiwifruit varieties. In this experiment, HT and HY kiwifruits were selected to observe phenotypic changes after Psa inoculation. As shown in Fig 1, when exposed to Psa, HY shoots exhibited clear symptoms: the inoculation area turned brown, the lesion length was about 2.26 mm at 12 h, and from 24 h to 144 h the lesion length gradually increased. In contrast, no lesions were found on HT shoots at any time point. Notably, another typical symptom was observed in the HY shoots 144 h after Psa infection; the inoculated shoots exhibited milky white bacterial pus. However, no similar symptom occurred in the HT shoots. These results indicate that the HY variety is susceptible while the HT variety is resistant to Psa infection.

### RNA-Seq data analysis and DEGs in response to Psa infection

**Fig 2:**
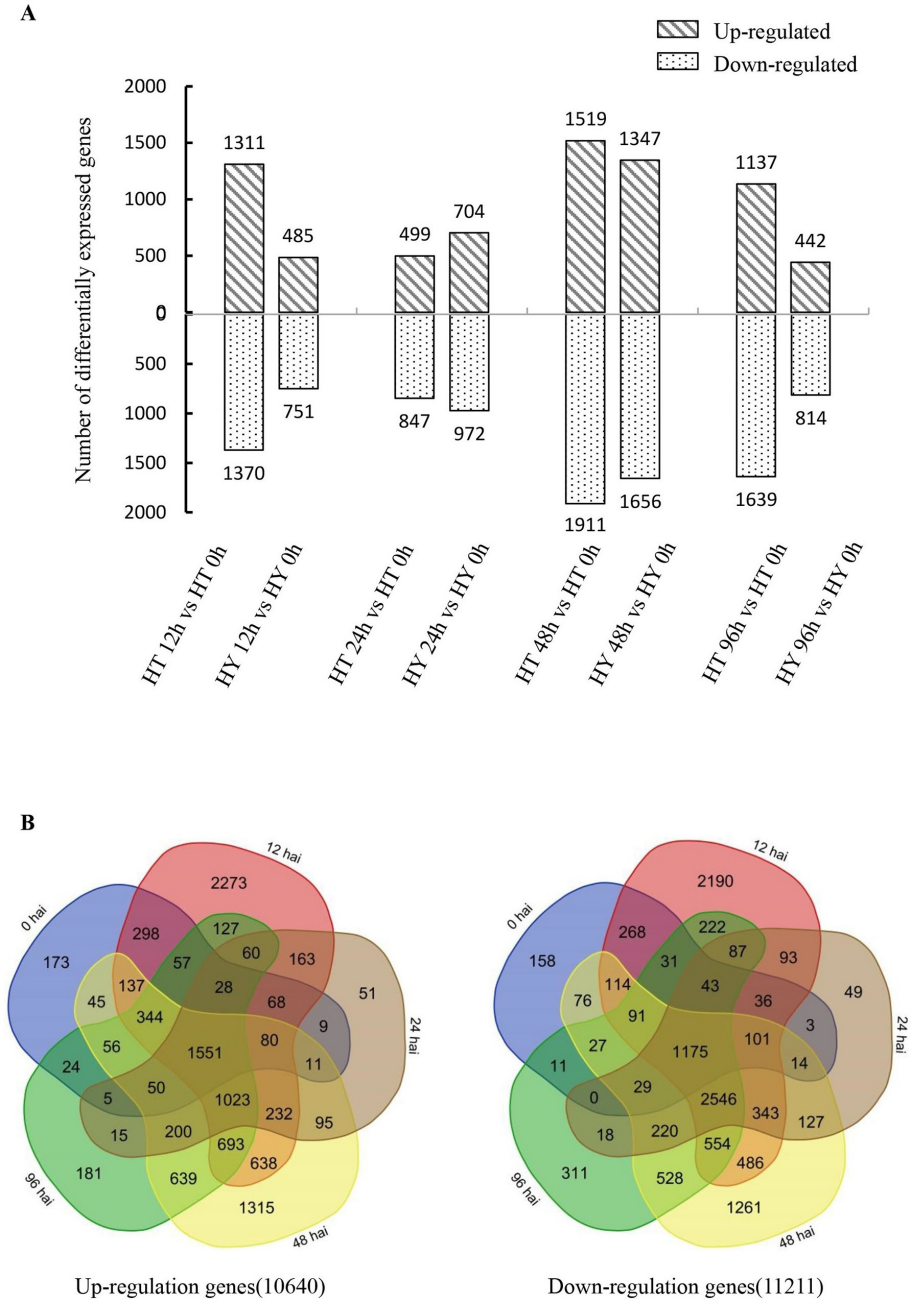
The number of the DEGs identified in Huate (HT) and Hongyang (HY) at 0, 12, 24, 48, and 96 hai with Psa. (A) Up- and down-regulated DEGs identified in comparisons between different time points in HT and HY. (B) DEGs were up- and down-regulated in HT compared with HY at 0, 12, 24, 48, and 96 hai. The total number of DEGs is at the bottom of each Venn diagram.

In order to determine the transcriptome profiles of HT and HY following Psa infection, we performed RNA-Seq analyses on HT and HY at 0, 12, 24, 48, and 96 hai. Three biological replicates were made at each time point for both cultivars. Pearson correlations between samples were used to calculate consistency. Pearson R^2^ varied from 0.83 to 1.0 (S1 Fig). The data demonstrated that the sequencing quality was sufficient for subsequent analysis. The raw reads varied from 46.24 to 84.35 million. After filtering and trimming, the clean reads were mapped from 42.77 to 77.82 million on the Kiwifruit Genome Database. Uniquely mapped reads accounted for 53.27% to 91.40% of the total mapped reads (Table 1). According to the total mapped reads, the expression levels of 45,729 genes were calculated using the FPKM method [22]. The number of DEGs between HT and HY was examined at 0, 12, 24, 48, and 96 hai with Psa (genes with P <0.05 found by DESeq were assigned as differentially expressed). Comparing HT12 (HT shoots at 12 hai) with HT0, the number of up-regulated genes and down-regulated genes was 1311 and 1370, respectively; comparing HT24 with HT0, the numbers were 499 and 847, respectively; comparing HT48 with HT0, 1519 up-regulated genes and 1911 down-regulated genes were identified; and comparing HT96 with HT0, 1137 up-regulated genes and 1639 down-regulated genes were identified. In the HY12 vs HY0 comparison, the number of up-regulated genes and down-regulated genes was 485 and 751, respectively; in the HY24 vs HY0 comparison, 704 DEGs were up-regulated and 972 DEGs were down-regulated. Comparing HY48 with HY0, there were 1347 up-regulated genes and 1656 down-regulated genes; and comparing HY96 with HY0 there were 442 up-regulated genes and 814 down-regulated genes. Overall, the number of DEGs (up and down-regulated) was significantly higher in HT than in HY at 12 hai (Fig 2A). These results suggest that more DEGs are involved in early disease resistance and a rapid response to pathogen infection in HT. In addition, the number of DEGs was also obviously higher in HT96, suggesting that most DEGs were related to a later defense response. Comparing HT and HY at all five time points, 10640 up-regulated genes and 11211 down-regulated genes were identified in HT. Between HT12 and HY12, the number of up-regulated genes and down-regulated genes was 7771 and 8379, respectively, which was significantly higher than that at any other time point. Overall, 12 hai was shown to be important for kiwifruit response to Psa infection (Fig 2B).

**Table 1:** Summary of RNA-Seq data.

Sample	Raw reads	Clean reads	Total mapped	Mapped reads (%)	Uniquely mapped
HY0_1	65507818	61766990	56179135	90.95%	54433558 (88.13%)
HY0_2	68801198	64509430	58633193	90.89%	56789252 (88.03%)
HY0_3	55080614	52213246	46790526	89.61%	45354363 (86.86%)
HY12_1	67232328	61085066	57033943	93.37%	55208799 (90.38%)
HY12_2	65293720	58699880	54565855	92.96%	52737406 (89.84%)
HY12_3	54996588	45697106	42953870	94.00%	41618981 (91.08%)
HY24_1	66347332	55213346	52160400	94.47%	50463541 (91.40%)
HY24_2	67135506	64498956	58603665	90.86%	56587917 (87.73%)
HY24_3	81454280	77828726	70679948	90.81%	68202854 (87.63%)
HY48_1	62503856	60301022	55807428	92.55%	53830782 (89.27%)
HY48_2	72589226	67329968	60424284	89.74%	58248444 (86.51%)
HY48_3	55596270	52644192	47144486	89.55%	45502131 (86.43%)
HY96_1	59299348	56036448	50178296	89.55%	48488514 (86.53%)
HY96_2	59538226	55615252	50256626	90.36%	48444877 (87.11%)
HY96_3	78104234	63063364	58727600	93.12%	56780950 (90.04%)
HT0_1	48024200	42771292	28555560	66.76%	27733383 (64.84%)
HT0_2	53105302	47203528	30158832	63.89%	29315098 (62.10%)
HT0_3	64694570	59514194	41845138	70.31%	40498510 (68.05%)
HT12_1	82986864	76706804	43359699	56.53%	41959042 (54.70%)
HT12_2	60672654	55730648	30961099	55.55%	29988614 (53.81%)
HT12_3	66170376	60243812	33112074	54.96%	32093440 (53.27%)
HT24_1	49280734	43813382	26070966	59.50%	25261905 (57.66%)
HT24_2	82936620	77170638	47606334	61.69%	46017020 (59.63%)
HT24_3	74247260	69136052	42881623	62.02%	41382843 (59.86%)
HT48_1	76081730	70383498	43208659	61.39%	41792793 (59.38%)
HT48_2	84351992	78356064	47444966	60.55%	45823191 (58.48%)
HT48_3	80053098	74106600	44987452	60.71%	43381559 (58.54%)
HT96_1	65446424	61951714	36168679	58.38%	34986281 (56.47%)
HT96_2	46240838	43245842	27265986	63.05%	26385042 (61.01%)
HT96_3	57419846	54552182	33523854	61.45%	32427681 (59.44%)

### Functional classification of DEGs after Psa inoculation

**Fig 3:**
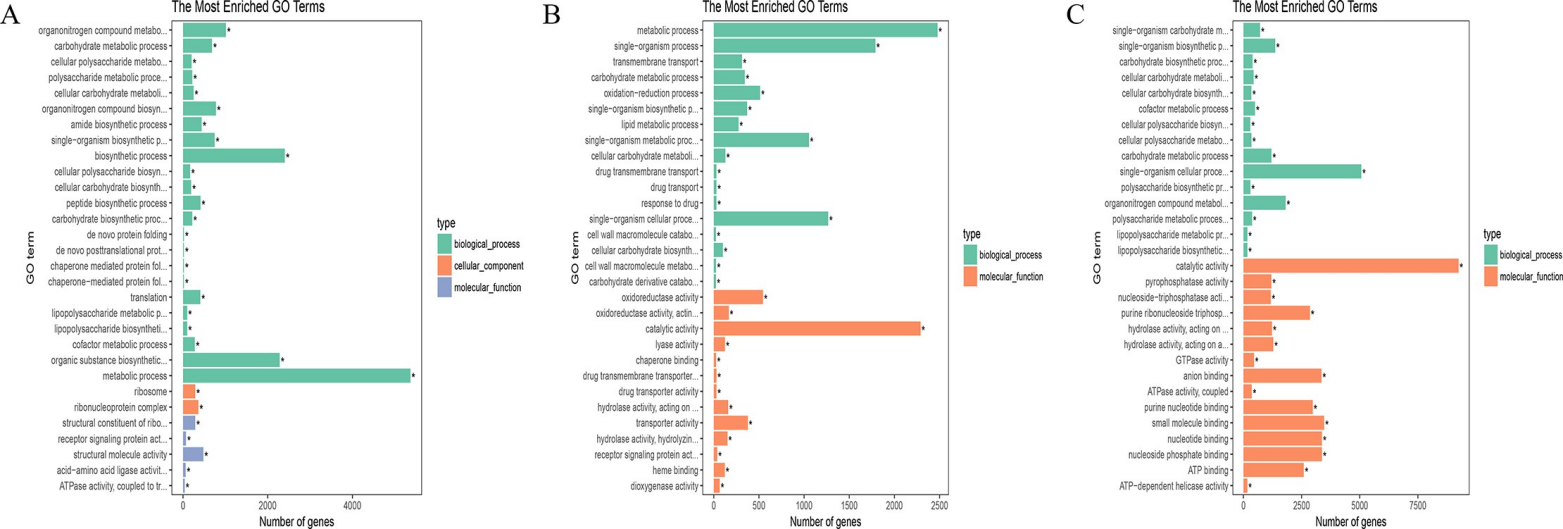
GO enrichment analysis of the DEGs in comparisons between Huate (HT) and Hongyang (HY) and between different time points after Psa infection. (A) GO enrichment analysis of HT at 12, 24, 48, and 96 hai with Psa compared with 0 hai. (B) GO enrichment analysis of HY at 12, 24, 48, and 96 hai compared with 0 hai. (C) GO enrichment analysis of HT and HY infected with Psa at the same time points.

We performed GO analysis to clarify the functions of DEGs in comparisons between the two cultivars and between different time points after Psa infection. GO can be divided into three categories: biological process, cellular component, and molecular function. DEGs from different time points in HT were found to be linked to biological processes, as the “metabolic process”, “organonitrogen compound metabolic process”, and “biosynthetic process” were significantly enriched in these comparisons (Fig 3A). For the DEGs from different time points in HY, within the biological process, “metabolic process” and “single-organism process” were significantly enriched, within the molecular function, “catalytic activity” was significantly enriched (Fig 3B). For the DEGs found between the two cultivars, within the biological process, “single-organism cellular process” and “organonitrogen compound metabolic process” were significantly enriched, within the molecular function, “catalytic activity”, “oxidoreductase activity”, and “pyrophosphatase activity” were significantly enriched (Fig 3C).

### Gene co-expression network analysis

**Fig 4:**
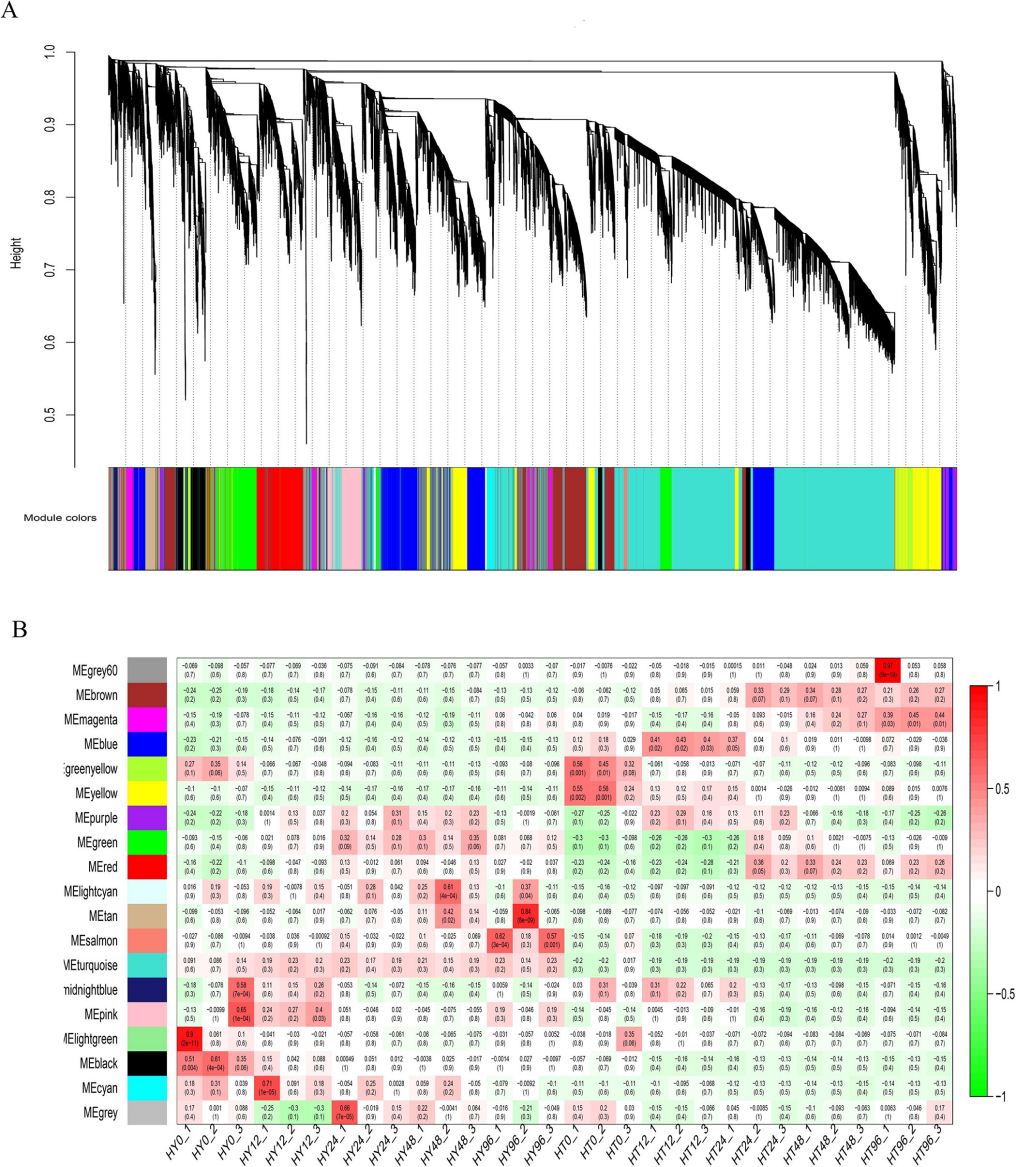
Gene co-expression network analysis with weighted gene co-expression network analysis (WGCNA). (A) Gene Dendrogram colored according to the correlations between gene expression levels. A total of 19 different modules were obtained. Different colors represent different gene modules on the abscissa and the ordinate indicates coefficients of dissimilarity between genes. (B) Module-sample association. The abscissa represents samples and the ordinate represents modules. The numbers in the cells are the correlation coefficient (top) and P-value (bottom). The variation from green (low) to red (high) indicates ranges of the DEGs.

**Fig 5:**
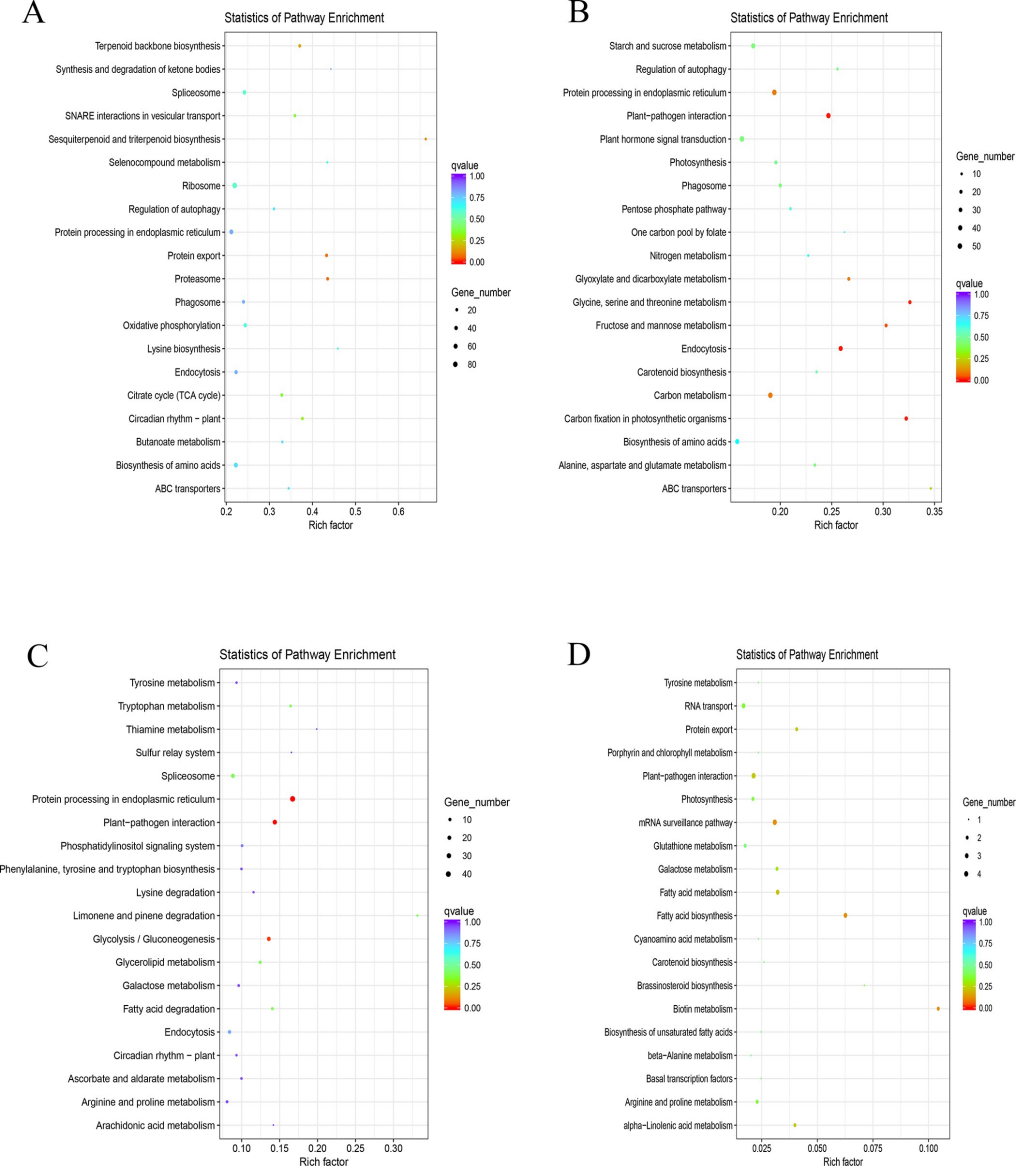
Genes in modules were analyzed by KEGG pathway. (A-D) represent MEblue, MEgreenyellow MEyellow, MEblack, and MEsalmon modules, respectively. the ordinate represents KEGG pathways and the abscissa represents Rich factor. Rich factor is the ratio of differentially expressed genes (DEGs) enriched in this pathway to the total number of annotated genes in the pathway. The size of the points indicates the number of DEGs in each pathway and the colors of the dots represent different p-values; the closer you get to red, the more significant the enrichment.

Weighted gene co-expression network analysis is a common algorithm for constructing gene co-expression networks [27]. A total of 19 different modules were obtained using a gene dendrogram colored according to the correlations between gene expression levels (Fig 4A). Among them, five modules were obviously differentially expressed between HT and HY. Genes in MEblue were highly expressed in HT12 while genes in MEgreenyellow and MEyellow had a high expression in HT0. In contrast, genes in MEblack were highly expressed in HY0, and genes in MEsalmon had a high expression level in HY96 (Fig 4B). We performed KEGG analysis for the five modules. Genes in MEgreenyellow and MEyellow had the same expression patterns, with high expression levels in HT0, therefore, we performed KEGG analysis on the genes of the two modules together. For the MEgreenyellow and MEyellow modules (Fig 5B), pathways related to“plant-pathogen interaction”,“Endocytosis”, “Glycine, serine and threonine metabolism”, and “Carbon fixation in photosynthetic organisms” were enriched, whereas for MEblack (Fig 5C), pathways related to “protein processing in endoplasmic reticulum”, “plant-pathogen interaction”, and “Glycolysis / Gluconeogenesis” were enriched.

### Identification of the genes associated with plant innate immune response

**Fig 6:**
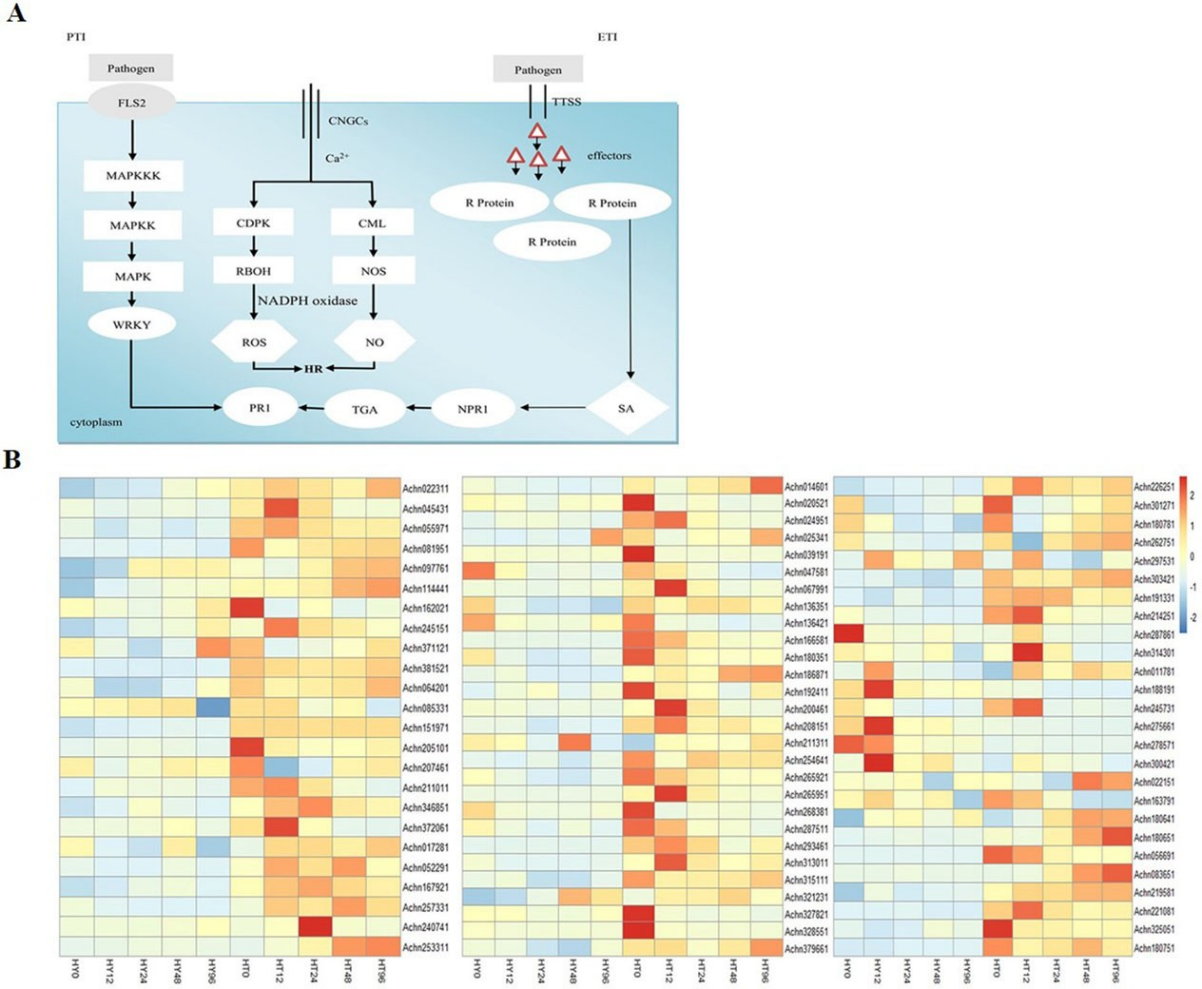
Expression patterns of the DEGs involved in PTI and ETI at different time points (0, 12, 24, 48, and 96 hai) in response to Psa infection in Huate (HT) and Hongyang (HY). (A) Schematic diagram of the plant’s innate immune system response to Psa inoculation. (B) Heat maps of DEGs involved in PTI and ETI at different time points (0, 12, 24, 48 and 96 hai) in response to Psa infection in HY and HT. Gene expression level was measured using the FPKM method (expected number of fragments per kilobase of transcript sequence per millions of base pairs sequenced). Horizontal axes represent gene ID, and vertical axes represent 0, 12, 24, 48, and 96 hai (from left to right) with Psa in HY and HT. Heat map shows the expression of genes ranging from blue (low expression) to red (high expression).

Plants face a variety of stresses throughout their lives. The plant innate immune system is able to identify pathogens and develop a defense response, which is divided into two main mechanisms: PTI and ETI [12,13]. In our study, a total of 42 DEGs were involved in the process (Fig 6A). Pathogen-associated molecular patterns are recognized by pattern recognition receptors that can activate PTI in the early stages. The signal is delivered to the cell through endocytosis, activating a series of protein kinases, such as the mitogen-activated protein kinase cascade, which is an important downstream part of PTI [28,29]. In *Arabidopsis*, flg22 is recognized by FLS2, and subsequently activates the mitogen-activated protein kinase cascade and the downstream transcription factor WRKY [30,31]. In our study, *FLS2-like* gene (*Achn180751*) was up-regulated at 0 hai in HT compared to HY. The expression of *MKK4/5-like* genes (*Achn226251* and *Achn301271*) were also much higher at 0 hai in HT. In addition, three *MEKK1-like* genes (*Achn180781*, *Achn262751*, and *Achn297531*) were also identified. WRKY transcription factors, which play a critical role in plant defense response, have both positive and negative regulators [32]. The six genes encoding WRKY22 initially exhibited low expression in both cultivars but, with the exception of *Achn245731*, their expression levels were relatively higher at 12 hai in HY compared to those in HT. The expression of *WRKY33-related* genes (*Achn191331*, *Achn214251*, *Achn287861*, and *Achn314301*) was also identified in response to Psa. The four genes were up-regulated in HT12 compared to HT0, while they were down-regulated in HY12. In addition, the expression levels were much higher in HT at 12 hai compared to those in HY (Fig 6B and S1 Table). Taken together, the *PTI-related* gene responses in the early stages after Psa infection (0 or 12 hai) might be an important response in HT against Psa.

Pathogens can also involve a series of effectors which then interact with resistance proteins to form the second stage of the plant immune system: ETI. Pathogen effectors activate ETI, resulting in plant disease resistance and a hypersensitivity response. This response can inhibit the growth, reproduction, and expansion of pathogenic bacteria [33,34]. Pto is a resistant protein against *Pseudomonas syringae* pv. *tomato*, which can recognize the pathogenic bacteria effector proteins AvrPtoB and AvrPto, triggering the downstream defense reaction, and eventually producing a hypersensitivity reaction at the infection site to prevent the colonization of pathogenic bacteria. Pti (Pto interaction protein) genes (Pti4 and Pti5) can further improve tomato resistance to Pst [35,36]. To clarify the role of ETI in the defense response after Psa infection, the expression levels of resistance protein-like genes were identified in the experiment. Five genes encoding Pti1 (*Achn056691*, *Achn083651*, *Achn219581*, *Achn221081*, and *Achn325051*) and four genes encoding RPS2 (*Achn022151*, *Achn163791*, *Achn180641*, and *Achn180651*) were expressed at low levels in HT and HY, although the difference between the two cultivars was considerable. The expression of *Pti1-related* genes was higher in HT compared to HY at all time points. The *RPS2-like* genes, except *Achn163791*, were more highly expressed in HT than in HY shoots at 48 and 96 hai. (Fig 6B and S1 Table). The patterns indicate that resistance genes played a critical role and that the ETI reaction was much stronger in HT than in HY.

### Activation of Ca^2+^ internal flow and respiratory burst oxidase homologs

Plant cyclic nucleotide-gated ion channels (CNGCs) are an important part in signal transduction cascades, which play an important role in plant hypersensitivity. These channels can participate in the regulation of Ca^2+^ internal flow [37,38]. Here, 10 genes encoding these nucleotide-gated ion channels were assessed. Most of the genes were more highly expressed in HT than in HY at 12 hai. In particular, *Achn0455431* and *Achn055971* were down-regulated at HY12 compared to HY0, while they were up-regulated at HT12 compared to HT0. The expression of the two genes was significantly higher at HT12. On one hand, Ca^2+^ combined with a calcium-binding protein (CML) produces NO, which further promotes plant hypersensitivity responses or autoimmune reactions [39]. In this study, eight homologs of CML were more highly expressed in HT than in HY at all time points. Notably, *Achn166581* was up-regulated more than five-fold in HT0, in addition, the expression of *Achn014601* was elevated ten-fold in HT compared with HY at 96 hai. On the other hand, Ca^2+^ can also activate calcium dependent protein kinases (CDPKs), and then phosphorylate downstream target proteins and activate respiratory burst oxidase homolog (RBOH) activity [40,41]. The main function of RBOH, also known as NADPH oxidase, is to produce reactive oxygen species [42,43], which play a critical role in plant responses to abiotic and biotic stresses. In the present study, changes in the expression of *CDPK-* and *ROBH-like* genes were observed in both cultivars in response to Psa infection. Eight genes of the homologs of CDPK were all up-regulated in HT compared with HY at all time points. Four genes encoding ROBH (*Achn017281*, *Achn052291*, *Achn167921*, and *Achn257331*) had low expression levels in both cultivars. In comparison, the expression of *ROBH-like* genes was higher in HT than in HY, in particular, *Achn052291* was quite high at 12 hai in HT (Fig 6B and S1 Table). Compared with previous studies, it is clear that Ca^2+^ participated in signal transduction, especially in the early response of Psa infection in kiwifruit and the expression level of ROS was higher in HT, which inhibited the infection of pathogen.

### DEGs involved in secondary metabolism

**Fig 7:**
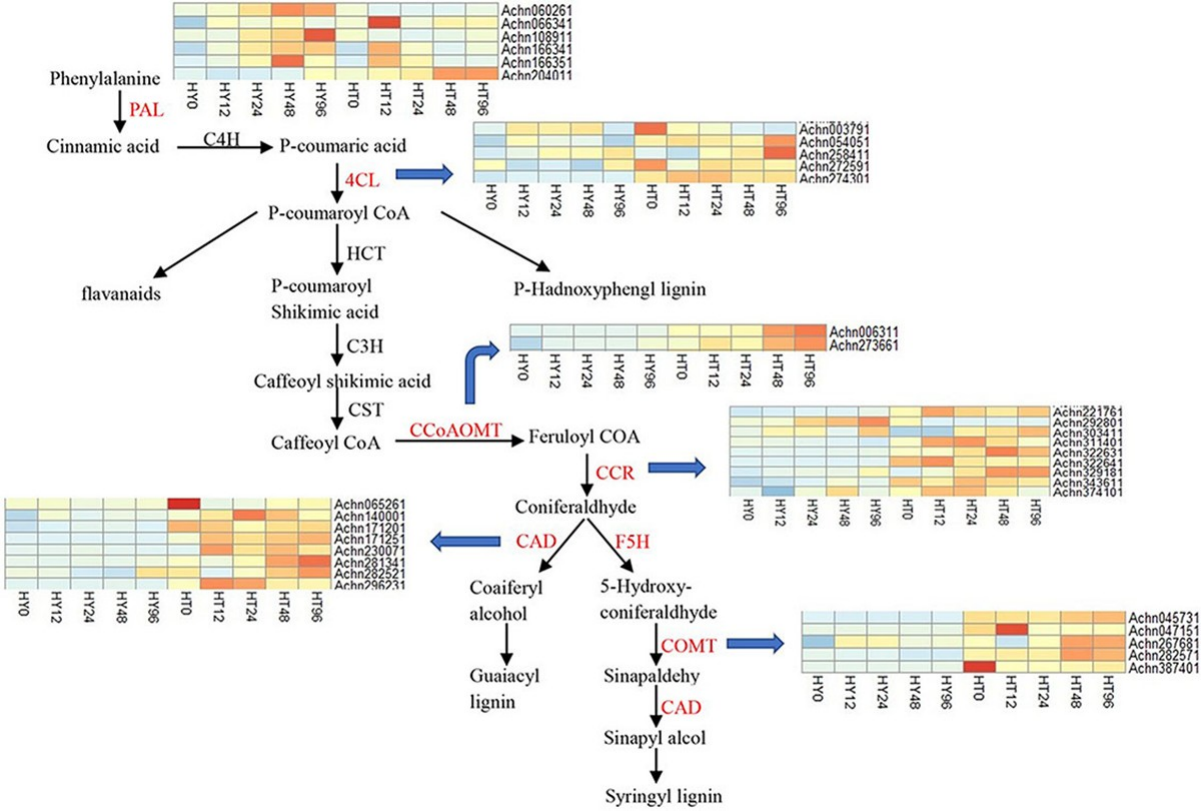
Expression patterns of the DEGs involved in the biosynthesis of phenylpropanoid at different time points (0, 12, 24, 48, and 96hai) in response to Psa infection in Huate (HT) and Hongyang (HY). Gene expression level was measured using the FPKM method (expected number of fragments per kilobase of transcript sequence per millions of base pairs sequenced). Heat map shows the expression of genes ranging from blue (low expression) to red (high expression). The expressed genes represented are phenylalanine ammonia-lyase (PAL), 4-hydroxycinnamoly CoA ligase (4CL), cinnamyl alcohol dehydrogenase (CAD), cinnamoyl-CoA reductase (CCR), caffeoyl-CoA O-methyltransferase (CCoAOMT), and caffeic acid 3-O-methyltransferase (COMT).

In general, secondary metabolites, such as lignin, play a critical role in the struggle between plants and pathogens. Studies have shown that when plants are infected by pathogenic bacteria, lignin is synthesized in large quantities at the infection site, strengthening the lignification of plant cell walls and resisting further infection by the pathogenic bacteria. The phenylpropanoid pathway mainly synthesizes lignin and flavonoids [44,45]. When *Gossypium hirsutum* was inoculated with pathogenic bacteria, a large amount of lignin was synthesized in the stem, which improved its resistance to verticillium wilt [46]. In our study, a total of 35 DEGs were involved in lignin biosynthesis, including phenylalanine ammonia-lyase (PAL) (6 genes), 4-coumarate CoA ligase (4CL) (5 genes), cinnamyl alcohol dehydrogenase (CAD) (8 genes), cinnamoyl-CoA reductase (CCR) (9 genes), caffeoyl-CoA O-methyltransferase (CCoAOMT) (2 genes) and caffeic acid 3-O-methyltransferase (COMT) (5 genes), As described in Fig 7, most of the genes were up-regulated in HT compared to HY after Psa infection. Notably, the expression levels of the *4CL-like* gene (*Achn274301*) and five homologs of CAD-like genes (*Achn296231*, *Achn282521*, *Achn281341*, *Achn171201*, and *Achn140001*) were significantly higher in HT. Three homologs of CCR (*Achn221761*, *Achn322631*, and *Achn343611*) were up-regulated more than ten-fold in HT compared to HY at 0 hai. In particular, the expression of the *CCR-like* gene (*Achn322641*) displayed a large difference between HT and HY at all time points, with a more than twenty-fold increase in HT. *CCoAOMT-like* genes (*Achn006311* and *Achn273661*) were higher at HT96 than at any other time points, *Achn045731* and *Achn387401* encoding COMT had a very low expression in HY whereas expression levels were much higher in HT. In addition, previous studies have reported that glutathione S-transferase (GST) played an important role in flavonoid biosynthesis [47,48]. In our experiment, most of the *GST-like* genes were significantly up-regulated in HT12. Of these genes, *Achn010311* and *Achn296121* were most highly expressed at 12 hai (S2 Fig and S1 Table).

### Crosstalk of phytohormone signaling pathways in plant defense responses

**Fig 8:**
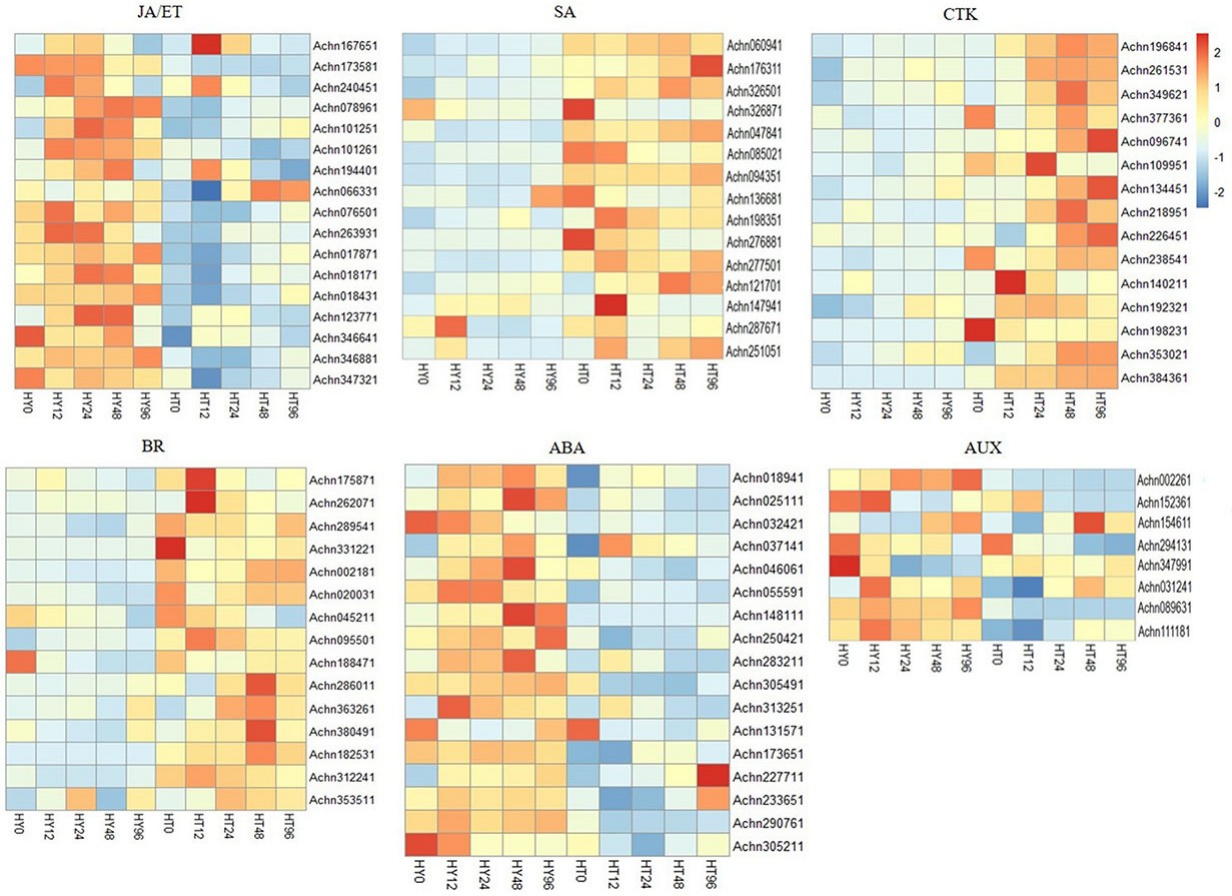
An overview of phytohormone signaling pathways after Psa inoculation, including the SA, JA, ET, AUX, CTK, BR, and ABA signaling pathways. Gene expression level was measured using the FPKM method (expected number of fragments per kilobase of transcript sequence per millions of base pairs sequenced). Horizontal axes represent gene ID and vertical axes represent 0, 12, 24, 48, and 96 hai (from left to right) with Psa in Hongyang (HY) and Huate (HT). Expression patterns of the DEGs range from blue (low expression) to red (high expression).

Plant pathogens are divided into three categories: biotrophs, hemibiotrophs, and necrotrophs, according to their lifestyles. It is clear that the SA signaling pathway mediates against biotrophic and hemibiotrophic pathogens to activate defense responses [49]. SA is a critical regulator in plant-pathogen interactions, and it can induce plant hypersensitive responses and systematic acquired resistance [50,51]. In our experiment, most of the DEGs involved in the SA signaling pathway (NPR1, TGA, and PR1) were up-regulated in HT after Psa infection. Importantly, the expression of the *TGA* gene (*Achn047841*), an important regulator acting downstream of the SA signaling pathway, interacting with NPR1 to positively regulate the expression of PR1 and the plant’s disease resistance response [52,53], was 6-fold higher in HT than in HY. In addition, we also found that the *PR1-like* gene (*Achn253311*) was up-regulated more than 5-fold at 12 hai, increasing to 17-fold at 96 hai in HT. Previous reports have suggested that acibenzolar-S-methyl, operating as a functional analogue of SA, is an effective inhibitor of Psa invasion in kiwifruit plants [18]. Isochorismate synthase is a pivotal enzyme responsible for SA accumulation [54]. *Achn121701*, with homology to isochorismate synthase, was up-regulated in HT (Fig 8 and S1 Table). All these results indicate an induced SA signaling pathway in HT after Psa infection. In contrast, the jasmonic acid (JA)/ethylene (ET) signaling pathway is mainly resistant to necrotrophic pathogens, and the SA and JA/ET signaling pathways are antagonistic to each other [55]. The genes involved in the JA/ET signaling pathway were identified in this study. The JA signaling component JAZ and ET signaling components ETR, EIN2, and EIN3 were highly expressed in HY compared to HT. Notably, *EIN2-related* genes (*Achn066331*, *Achn076501*, and *Achn263931*) were up-regulated in HY but down-regulated in HT at 12 hai after Psa infection. The transcription factor WRKY70 is a common component in the SA- and JA-mediated signaling pathways: WRKY70 is an activator in the SA signaling pathway but an inhibitor in the JA signaling pathway [56]. In the present experiment, *WRKY70-like* genes (*Achn287671* and *Achn251051*) were highly expressed in HT compared to HY (Fig 8 and S1 Table). These results indicate that the SA signaling pathway, but not the JA/ET signaling pathway, played a pivotal role in the defense response of HT shoots after Psa inoculation, to improve kiwifruit resistance.

It has also been reported that auxin is involved in the plant disease resistance response. The auxin level of *Arabidopsis* lacking a functional RPS2 gene (rps2 mutant), is increased after infection with *P*. *syringae* pv. *tomato* strain DC3000 (PstDC3000); the resistance of the rps2 mutant is also decreased [57]. The resistance of the auxin-insensitive axr2-1 *Arabidopsis* mutation is improved during PstDC3000 infection, suggesting that the auxin signaling pathway is harmful to the *Arabidopsis* immune response to PstDC3000 infection [58]. In our study, most of the genes (5 *AUX1* and 3 *TIR1*) involved in the AUX signaling pathway were down-regulated in HT shoots compared to HY shoots. Interestingly, the *TIR1* gene (*Achn089631*) had a strong down-regulation in HT12 (Fig 8 and S1 Table). In addition, Yasuda reported an antagonistic interaction between the SA- and abscisic acid (ABA)-mediated signaling pathways in *Arabidopsis* [59]. Indeed, 17 genes (11 *PYR/PYL* and 6 *ABF* genes) related to the ABA signaling pathway were down-regulated in HT shoots compared with HY shoots at all time points (Fig 8 and S1 Table). All the studies suggested that AUX and ABA might have negative regulatory effects during Psa infection.

Cytokinins modulate the SA signaling pathway to promote the resistance of *Arabidopsis* to *Pseudomonas syringae* pv. *tomato* DC3000 (Pst) [60]. In addition, wild-type tobacco resistance to the viral pathogen tobacco mosaic virus and the bacterial pathogen *Pseudomonas syringae* pv. *tabaci* (Pst) can be enhanced by brassinosteroids (BRs) [61]. The expression patterns of *CTK- and BR-related* genes were identified in our study. The genes involved in the CTK signaling pathway, for example *AHK-like* genes and *ARR-like* genes, were up-regulated in HT. In addition, the genes encoding the BR signaling pathway were also up-regulated in HT, as a result of the kiwifruit plant’s response to Psa. (Fig 8 and S1 Table).

Taken together, these results indicate that the SA signaling pathway played a positive role in the early stages of HT against Psa. The DEGs involved in the SA-, CTK- and BR- signaling pathways were up-regulated in HT shoots, whereas the most of genes involved in the JA/ET-, ABA-, and AUX-signaling pathways were down-regulated in HT shoots. The different expression patterns of multiple phytohormone signaling pathways indicated that phytohormone signaling pathways are not independent of each other, but that there is crosstalk amongst them.

### Verification of gene expression levels by qRT-PCR

**Fig 9:**
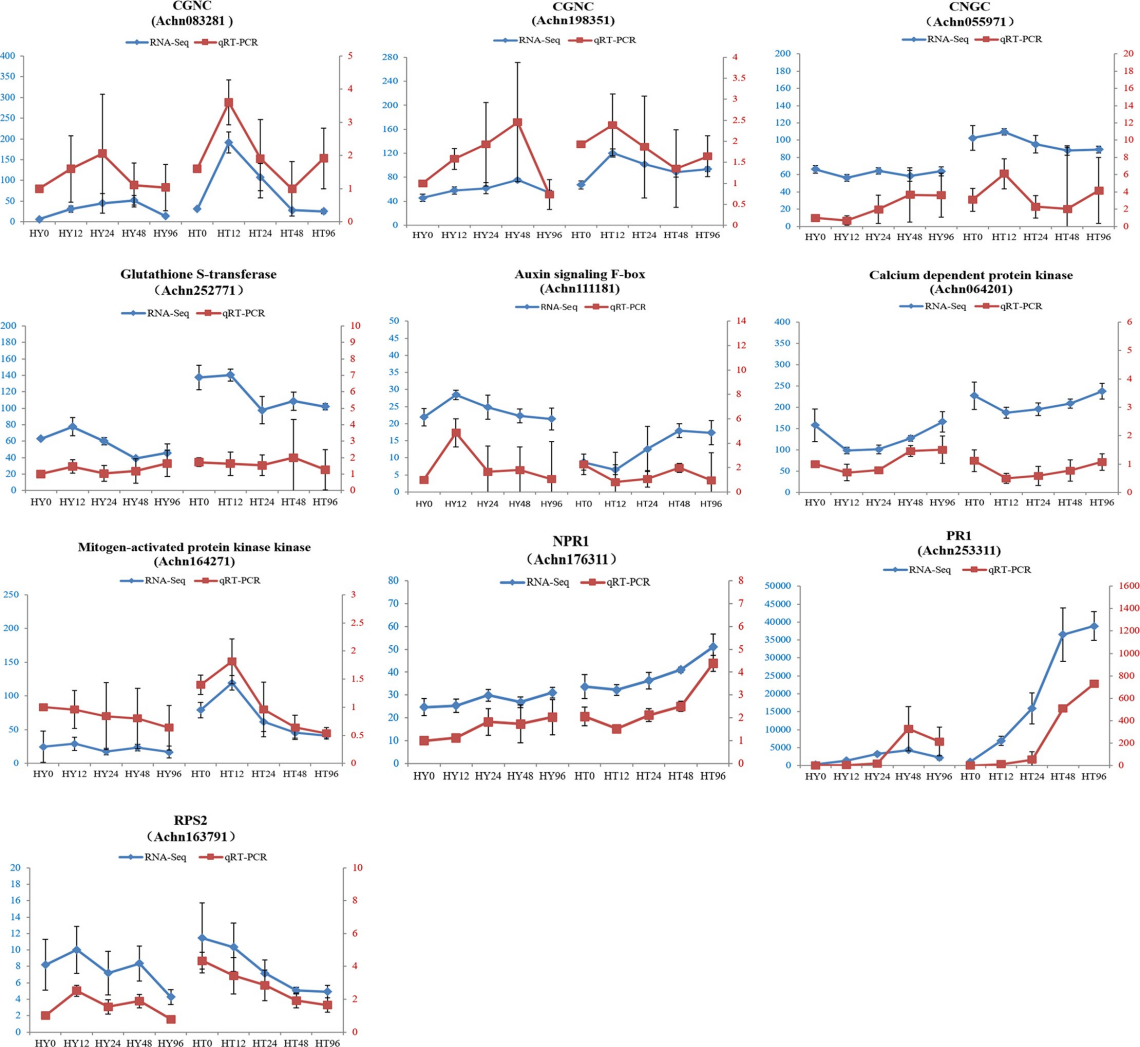
Relative expression levels of ten selected genes at 0, 12, 24,48, and 96hai with Psa in Huate (HT) and Hongyang (HY) measured using qRT-PCR. Left ordinate (in blue) represents the FPKM (expected number of fragments per kilobase of transcript sequence per millions of base pairs sequenced) of RNA-Seq. Right ordinate (in red) represents the relative expression level of qRT-PCR. The abscissa represents 0, 12, 24, 48, and 96 hai (from left to right) with Psa in HY and HT.

To confirm the validity of the RNA-Seq, ten DEGs were randomly chosen for qRT-PCR. The expression data of the qRT-PCR were consistent with the RNA-Seq results at all time points, indicating a similar trend between transcriptome analysis and qRT-PCR data. The primers of DEGs are listed in S2 Table, and the qRT-PCR data are shown in Fig 9.

## Conclusion

In this study, transcriptome analysis was performed on resistant HT and susceptible HY at 0, 12, 24, 48 and 96 hai with Psa. Some DEGs related to disease resistance were identified that were involved in plant innate immune response, Ca^2+^ internal flow, secondary metabolism, and phytohormone signaling pathways. This study provides important information to elucidate the defense molecular mechanisms of kiwifruits during Psa infection.

## Supporting information

S1 Fig Pearson correlation between samples. (TIF)

S2 Fig The expression patterns of differentially expressed genes (DEGs) involved in glutathione S-transferase (GSTs) at different time points in response to *Pseudomonas syringae* pv. *actinidiae* in Huate and Hongyang kiwifruit shoots. (TIF)

S1 Table Information of differentially expressed genes between Huate and Hongyang kiwifruit shoots. (XLS)

S2 Table Primers used for quantitative real-time RT-PCR experiments. (XLS)
