# Supplementary figures and images for: Comparative transcriptome analysis of resistant and susceptible kiwifruits in response to Pseudomonas syringae pv. Actinidiae during early infection
Source: PLoS One. 2019 Feb 19;14(2):e0211913. doi: 10.1371/journal.pone.0211913 (PMC6380551; doi:10.1371/journal.pone.0211913)

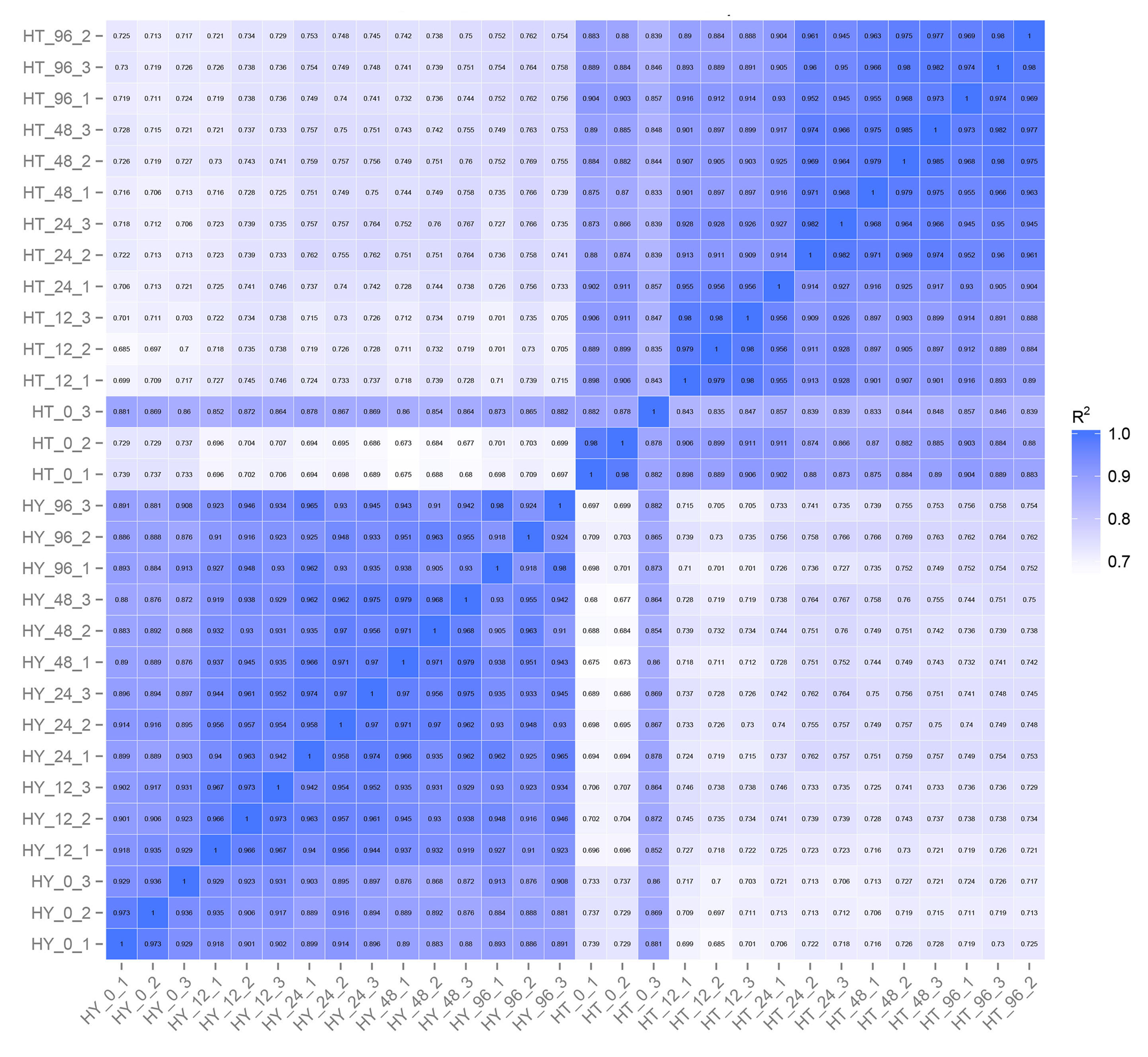

Supplement: S1 Fig — (TIF) [file pone.0211913.s001.tif]
